# Diagnostic concordance of GeneXpert HCV VL fingerstick and GeneXpert HCV viral load with RT-PCR in POCT settings among hemodialysis patients

**DOI:** 10.1371/journal.pone.0324671

**Published:** 2026-02-13

**Authors:** Andri Sanityoso Sulaiman, Pringgodigdo Nugroho, Darlene Raudhatul Bahri, Desti Rachmani

**Affiliations:** 1 Division of Hepatobiliary, Department of Internal Medicine, Faculty of Medicine Universitas Indonesia - Dr. Cipto Mangunkusumo Hospital, Jakarta, Indonesia; 2 Division of Nephrology and Hypertension, Department of Internal Medicine, Faculty of Medicine Universitas Indonesia - Dr. Cipto Mangunkusumo Hospital, Jakarta, Indonesia; Stellenbosch University, SOUTH AFRICA

## Abstract

**Background:**

Hepatitis C virus (HCV) remains a significant global health concern, particularly among high-risk populations such as patients undergoing hemodialysis. Although nucleic acid testing (NAT) using RT-PCR remains the gold standard for HCV RNA detection, its centralized laboratory workflow limits accessibility. Point-of-care molecular testing (PoCT), such as the GeneXpert Fingerstick assay, offers a decentralized alternative for HCV RNA quantification. However, its analytical concordance with reference methods in Indonesian settings remains limited.

**Methods:**

This study was a cross-sectional analytical concordance study involving 57 patients with chronic kidney disease (CKD) undergoing hemodialysis at Cipto Mangunkusumo and Pelni Hospitals in Jakarta. Each participant underwent HCV RNA testing using three methods: GeneXpert HCV VL Fingerstick, GeneXpert HCV VL Plasma, and Cobas TaqMan RT-PCR (as the reference standard). Agreement among methods was assessed using sensitivity, specificity, positive predictive value (PPV), negative predictive value (NPV), and Cohen’s κ coefficient. Correlation for quantifiable values was evaluated using linear regression analysis.

**Results:**

Both GeneXpert methods showed perfect concordance with the Cobas TaqMan RT-PCR, with sensitivity, specificity, PPV, and NPV all at 100%. Agreement analysis yielded a Cohen’s κ of 1.000 (*p* < 0.001) for both comparisons. Quantitative correlation between methods was also strong (*R²* > 0.95).

**Conclusion:**

The GeneXpert HCV VL Fingerstick assay demonstrates excellent analytical agreement with conventional molecular testing and may serve as a simple, practical, decentralized alternative for monitoring HCV RNA in hemodialysis patients.

## Introduction

Hepatitis C virus (HCV) is a major driver of liver-related morbidity and mortality, primarily through its progression to cirrhosis and hepatocellular carcinoma (HCC). According to the World Health Organization (WHO), HCV caused approximately 242,000 deaths globally in 2022, with most attributed to cirrhosis and HCC—the most common form of primary liver cancer. The WHO recommends a two-step diagnostic algorithm for hepatitis C: initial screening using an anti-HCV antibody test, followed by confirmatory testing for active infection using HCV RNA or core antigen assays. Point-of-care molecular tests like the GeneXpert Fingerstick are endorsed for use in decentralized settings, provided they meet a minimum limit of detection (LOD) of 1000 IU/mL to facilitate timely diagnosis and linkage to care [1]. At the global level, the burden of HCV is particularly pronounced among patients receiving dialysis. A recent systematic review and meta-analysis by Kenfack-Momo et al. reported a pooled global prevalence of 7.9% (95% CI: 6.7–9.3%) of HCV among dialysis patients, with higher rates in low- and middle-income countries compared to high-income settings. The same analysis found a pooled case fatality of 10.5% (95% CI: 6.7–15.9%), underscoring the excess risk faced by this population. Prevalence estimates ranged from as low as 3–4% in Western Europe and North America to over 20% in certain parts of Africa and Asia, reflecting wide geographical disparities in infection control and diagnostic access. These findings emphasize that dialysis patients are disproportionately affected by HCV not only due to repeated vascular access and potential nosocomial transmission, but also because of immunological vulnerability and cumulative comorbidities. [2]. In Indonesia, national data reflect this global pattern. The 13th Indonesian Renal Registry (IRR) reported that in 2020, 6,244 hemodialysis patients (18% of those tested) were anti-HCV reactive. Yet confirmatory HCV RNA testing is not routinely available in all dialysis centers. In many areas, patients must be referred to tertiary hospitals or centralized laboratories, highlighting the uneven distribution of molecular diagnostic facilities across the country. These limitations underscore the need for accurate and accessible viral load testing within dialysis units, where reliance on venous sampling and centralized NAT may delay diagnosis and treatment [3]. These data emphasize the importance of accurate and accessible viral load testing in dialysis units, where reliance on venous sampling and centralized NAT testing often presents logistical challenges.Consensus statements have long emphasized that molecular confirmation of HCV infection is essential to guide treatment initiation and monitoring, particularly in immunocompromised populations such as dialysis patients [4].Regional consensus statements, such as those from the Asian Pacific Association for the Study of the Liver (APASL), reinforce the need for confirmatory HCV RNA testing after antibody screening, particularly in immunocompromised populations such as dialysis patients, where antibody assays may be less sensitive [5]. Moreover, a recent meta-analysis encompassing more than 400 studies estimated the pooled prevalence of HCV among hemodialysis patients at 20.7% (95% CI 18.9–22.6) and confirmed that HCV infection was associated with a significantly increased risk of all-cause mortality in this population [6]. A meta-analysis identified several contributing factors, including the use of shared dialysis equipment, inadequate infection control measurement, and frequent blood sampling. Early diagnosis and timely management of HCV infection in this population are crucial to prevent complications such as cirrhosis and HCC [7]. However, conventional molecular testing often requires centralized laboratory facilities and venous sampling, which can delayed diagnosis and limited accessibility, particularly in hemodialysis settings. Point-of-care testing (PoCT) has gained increasing attention as a strategy to improve the diagnosis and management of HCV infection among hemodialysis patients. These assays, which can be performed at the bedside or within dialysis units, offer several advantages over conventional laboratory-based testing, including shorter turnaround times, simplified sample collection, and improved patient engagement. Among currently available PoCT platforms for HCV RNA detection, the GeneXpert system (Cepheid, USA) stands out as a particularly promising tool, combining full automation, minimal operator involvement, and adaptability to different specimen types. The GeneXpert HCV VL Plasma assay is a rapid, fully automated molecular test that enables direct detection and quantification of HCV RNA from plasma samples with high sensitivity and specificity. Although its quantitative results can support disease monitoring and treatment evaluation, its clinical utility may be limited in resource-constrained settings. The GeneXpert HCV VL Fingerstick assay is a point-of-care molecular test designed to enable same-day clinical decision-making by detecting HCV RNA from just 100 µL of capillary whole blood, thereby eliminating the need for follow-up visits [8].A real-world study conducted in Catalonia, Spain, demonstrated the effectiveness of the GeneXpert HCV Fingerstick assay as a single-step RNA-based PoCT among people who inject drugs, achieving a sensitivity of 98% and specificity of 100%. These findings highlight the potential of the GeneXpert HCV Fingerstick platform to overcome persistent barriers to HCV diagnosis, particularly in marginalized populations with limited access to centralized molecular testing [9]. In Germany, Petroff et al evaluated the feasibility of implementing the GeneXpert HCV Fingerstick assay in routine primary care settings. Conducted as part of a national preventive health check-up program, the study demonstrated that the assay could be effectively integrated into clinical workflows, yielding a diagnostic sensitivity of 95.5% and specificity of 98.1%. Although approximately 12% of tests produced invalid results mainly due to difficulties in obtaining sufficient capillary blood, the findings underscored the assay’s potential to support decentralized HCV diagnosis in primary care, particularly where access to centralized laboratories is limited [10]. Additional evidence supports the GeneXpert assay’s diagnostic accuracy across diverse clinical contexts. Beyond Europe, several studies from low- and middle-income countries have further validated the diagnostic accuracy of the GeneXpert platform across diverse clinical and epidemiological settings. Umumararungu et al. (2017) assessed its use among patients at a military hospital in Rwanda while Tang et al. (2022) evaluated its application in decentralized care settings, including HCV clinics and substance use treatment centers [11,12]. In both studies, the diagnostic accuracy of the GeneXpert assay was compared against established laboratory-based molecular assays, such as the Abbott RealTime HCV assay. Similarly, a field-based evaluation by Lamoury et al. reported that the Fingerstick assay achieved 98.3% sensitivity and 100% specificity for detecting HCV RNA from capillary blood among people who inject drugs in Australia, with minimal viral load deviation (mean difference −0.07 log IU/mL), reinforcing its reliability in field-based settings [13]. Furthermore, a study by Iwamoto et al. compared the diagnostic performance of GeneXpert (Cepheid) with Cobas TaqMan (Roche) in Cambodia, where genotypes 1 and 6 are predominant. Among 454 patients (77%) with detectable and quantifiable viral loads using the Roche assay, 195 (43%) were identified as genotype 6. The GeneXpert assay demonstrated 100% sensitivity (95% CI: 99.2–100.0) and 98.5% specificity (95% CI: 94.8–99.8), providing same-day results with an error rate of only 1%, compared with a four-day turnaround with the Roche assay [14].

## Materials and methods

### Study design

This study was a cross-sectional analytical concordance study designed to evaluate the diagnostic performance and agreement of the GeneXpert HCV VL Fingerstick assay for the quantitative detection of HCV RNA in patients with chronic kidney disease (CKD) undergoing hemodialysis. This population included both anti-HCV–positive and –negative individuals. The GeneXpert HCV VL Fingerstick assay was compared with the GeneXpert HCV VL Plasma assay and the Cobas TaqMan RT-PCR (Roche), which served as the reference gold standard. All GeneXpert-based tests were performed using the GXHCV-VL-CE-10 cartridge (Cepheid, Sunnyvale, USA) on a six-color, four-module GeneXpert R2 system (GXIV-4-L System, 900−0513) equipped with the GeneXpert Dx v4.6a software [15]. For additional comparison, selected plasma samples were also analyzed using the Abbott RealTime HCV Viral Load assay following the manufacturer’s protocol.

### Patients

**Fig 1:**
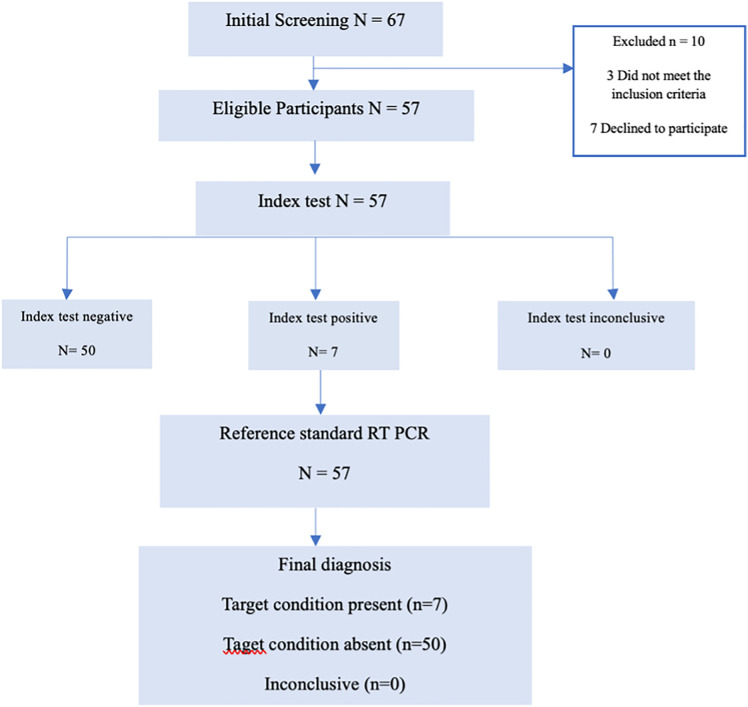
Participant flow diagram according to STARD 2015 recommendations. Enrollment, exclusions, and allocation for diagnostic testing.

A total of 57 patients with CKD undergoing maintenance hemodialysis were recruited from Cipto Mangunkusumo National Hospital and Pelni Hospital, Jakarta. Inclusion criteria were: (i) adults ≥18 years of age, (ii) undergoing maintenance hemodialysis, and (iii) willing to provide informed consent. Exclusion criteria were: (i) hepatitis B virus (HBV) infection, human immunodeficiency virus (HIV) infection, or the presence of hepatocellular carcinoma (HCC), and (ii) evidence of ascites. Baseline demographic and clinical data, including age, sex, and HCV antibody status were recorded. The study was approved by the Ethics Committee of the Faculty of Medicine, Universitas Indonesia (approval number KET-779/UN2.F1/ETIK/PPM.00/02/2022). Written informed consent was obtained from all participant prior to enrollment. Sample collection and diagnostic testing including fingerstick capillary sampling, plasma-based viral load measurement, and RT-PCR—were conducted between December 5, 2022 and July 1, 2023. (Fig 1)

### GeneXpert HCV VL fingerstick

Capillary whole blood (100 μL) was obtained from the fingertip using the BD Microtainer™ Contact-Activated Lancet, collected into an EDTA-coated minivette (Sarstedt Minivette PoCT), and immediately loaded into a GeneXpert HCV VL FS cartridge (GX-HCV-FS-CE-10; Cepheid). The cartridge, designed specifically for capillary whole blood, has a limit of quantification of 100 IU/mL and a limit of detection of 40 IU/mL. It was then inserted into the GeneXpert instrument, and results were generated within 60 minutes. This assay is simple to use, requires minimal training, and is well suited for point-of-care settings. In parallel, venous blood samples were processed using the GeneXpert HCV VL Plasma assay to provide a direct comparison with the fingerstick method.

### GeneXpert HCV VL plasma assay *(Venous blood sample)*

Venous blood samples were collected, and 1 mL of plasma was obtained after centrifugation. The plasma was then loaded into the GXHCV-VL-CE-10 cartridge and analyzed using the GeneXpert system. The assay required approximately 105 minutes to complete. The GeneXpert system, which consists of four independent modules connected to a laptop and a portable device, allows multiple cartridges to be processed simultaneously, ensuring an efficient workflow.

### Reference standard: Cobas TaqMan RT-PCR

The COBAS AmpliPrep/COBAS TaqMan assay (Roche, Ref. No. 05532264) was used as the reference standard in this study. A total of 750 μL of plasma was processed and tested according to the manufacturer’s protocol. Plasma samples obtained by venipuncture were centrifuged within 6 hours of collection and stored at −20 °C prior to analysis. The assay’s lower limit of detection (LOD) and ower limit of quantification (LLOQ) are comparable to those of other molecular methods used in this study.The Cobas TaqMan assay consists of three main steps (i) RNA plasma isolation, (ii) reverse transcription to complementary DNA (cDNA), (iii) PCR amplification and detection using dual-labeled oligonucleotide probes. These steps target a highly conserved region within the 5′ untranslated region of the HCV genome. The optimized nucleotide sequences allow for reliable amplification across all major HCV genotypes. All testing was performed with analysts blinded to the RT-PCR reference results to minimize the risk of observer bias.

### Statistical analysis

Data from the GeneXpert HCV VL Fingerstick and Plasma assays were analyzed independently. Diagnostic performance was evaluated by calculating sensitivity, specificity, positive predictive value (PPV), and negative predictive value (NPV). Concordance between assays was assessed using Cohen’s κ coefficient, and correlation of quantifiable viral load measurements was evaluated using linear regression, with the squared correlation coefficient (R²) reported. Only qualitative outcomes (detectable vs. non-detectable) were used for sensitivity and specificity analyses. The sample size of 57 patients was determined by convenience sampling. A post-hoc power analysis was conducted using G*Power version 3.1.9.7. Based on the observed Pearson correlation coefficient (r = 0.96) between the GeneXpert assays and the RT-PCR reference method, with α = 0.05 and n = 57, the computed statistical power (1–β) was 1.000, confirming that the study was adequately powered. All statistical analyses were performed using IBM SPSS Statistics version 26.0 A p-value < 0.05 was considered statistically significant. Invalid test results were excluded from the final analysis.

## Results

### Patient characteristics

A total of 57 patients with CKD undergoing maintenance hemodialysis were enrolled between December 2022 and July 2024. The cohort had a mean age of 52.88 ± 14.04 years (range:24–78 years), and 66.7% were male. Thirty-six patients (63.2%) were HCV antibody–positive while twenty-one (36.8%) were anti-HCV negative (Table 1).

**Table 1:** Baseline Characteristic.

Characteristic	Patient Number (n-57)
Sex, n(%)	
-Male	38 (66.7)
-Female	19 (33.3)
Age (mean ± SD)	52,88 ± 14.04
HCV antibody, n(%)	
-Reactive	36 (63.2)
-Non-reactive	21 (36.8)

### Concordance between the assays

**Fig 2:**
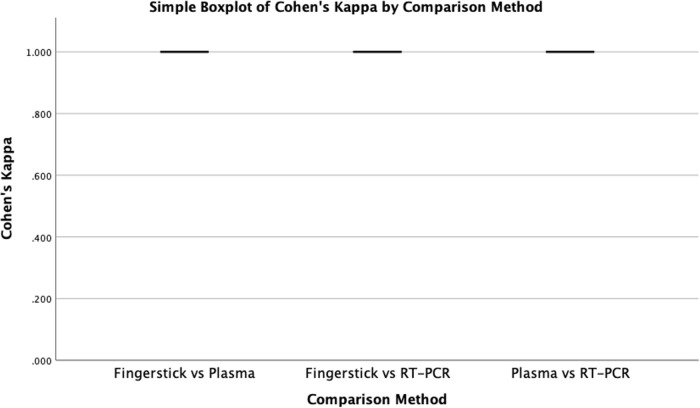
Cohen’s kappa values for pairwise qualitative agreement between GeneXpert HCV RNA measurements obtained from fingerstick and plasma samples and Cobas TaqMan 96 RT-PCR.

Among 57 patients, seven tested positive by all three methods: GeneXpert HCV VL Fingerstick, GeneXpert HCV VL Plasma, and Cobas TaqMan RT-PCR. Both GeneXpert assays demonstrated statistically significant concordance (p < 0.001; Tables 2, 3, 4), which was further supported by Cohen’s kappa (κ = 1.000; Fig 2). Although the sample size was limited, the perfect concordance observed highlights the reliability of the assays under the study conditions.

**Table 2:** Qualitative Result of Viral Load in Gene Xpert Fingerstick and Cobas Taqman.

GeneXpert Fingerstick	Cobas Taqman Viral load		Total n (%)	*p-Value*
	Virus detected n (%)	Virus not detected n (%)		
Virus detected	7 (100)	0 (0)	7 (100)	<0.001
Virus not detected	0 (0)	50 (100)	50 (100)	

Fingerstick sensitivity = 100%.

Fingerstick specificity = 100%.

Fingerstick PPV = 100%.

Fingerstick NPV = 100%.

**Table 3:** Qualitative viral load results comparing GeneXpert HCV VL (plasma) and Cobas TaqMan 96 RT-PCR.

GeneXpert	Cobas Taqman Viral load		Total n (%)	*p-Value*
	Virus detected n (%)	Virus not detected n (%)		
Virus detected	7 (100)	0 (0)	7 (100)	<0.001
Virus not detected	0 (0)	50 (100)	50 (100)	

Genexpert sensitivity = 100%.

Genexpert specificity = 100%.

Genexpert PPV = 100%.

Genexpert NPV = 100%.

**Table 4:** Qualitative viral load results comparing GeneXpert HCV VL and Cobas TaqMan 96 RT-PCR.

Fingerstick	GeneXpert		Total n (%)	*p-Value*
	Virus detected n (%)	Virus not detected n (%)		
Virus detected	7 (100)	0 (0)	7 (100)	<0.001
Virus not detected	0 (0)	50 (100)	50 (100)	

Genexpert sensitivity = 100%.

Genexpert specificity = 100%.

Genexpert PPV = 100%.

Genexpert NPV = 100%.

### Correlation between viral load measurements

**Fig 3:**
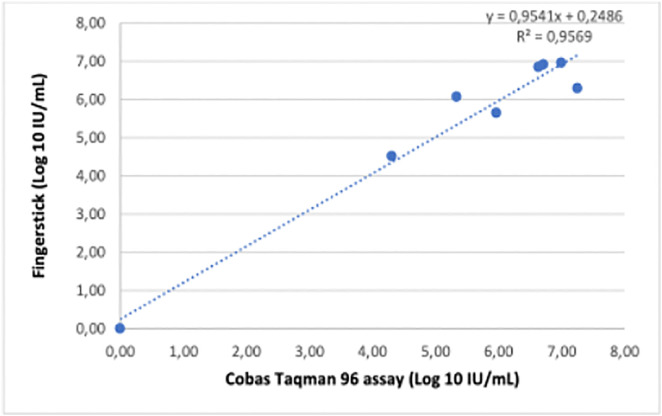
Linear regression analysis of GeneXpert HCV RNA (fingerstick) versus Cobas TaqMan 96 RT-PCR. Scatter plot showing the regression line and coefficient of determination (R²).

**Fig 4:**
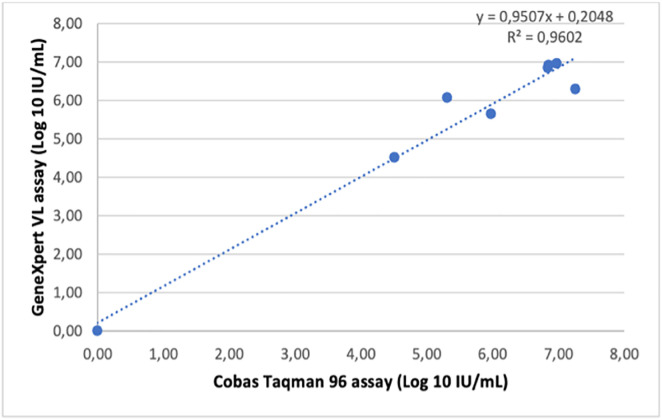
Linear regression analysis of GeneXpert HCV RNA (plasma) versus Cobas TaqMan 96 RT-PCR. Scatter plot showing the regression line and coefficient of determination (R²).

**Fig 5:**
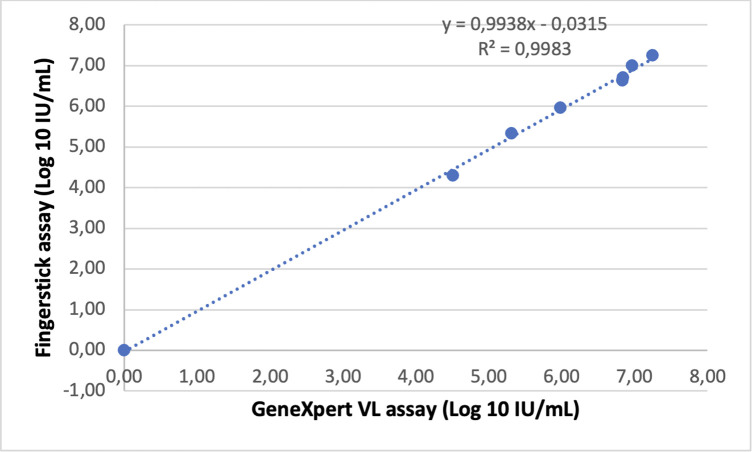
Linear regression analysis of GeneXpert HCV RNA (fingerstick) versus GeneXpert HCV RNA (plasma). Scatter plot showing the regression line and coefficient of determination (R²).

Linear regression of quantifiable viral loads measurements showed strong correlation between the GeneXpert HCV VL Fingerstick assay Cobas TaqMan (R = 0.9569, p < 0.001; Fig 3), as well between the the GeneXpert HCV Viral Load assay and Cobas Taqman 96 (R = 0.9602, p = 0.001; Fig 4). An exceptionally strong correlation was observed between the GeneXpert HCV VL Fingerstick and GeneXpert HCV VL Plasma assays (R = 0.9983, p < 0.001) (Fig 5). Sensitivity, specificity, PPV, and NPV were all 100%.

## Discussion

This study demonstrated that the GeneXpert HCV VL Fingerstick assay provides diagnostic performance equivalent to the GeneXpert HCV VL and Cobas TaqMan RT-PCR assays in patients undergoing maintenance hemodialysis. In practical terms, a capillary-blood approach can deliver the same qualitative agreement suitable for clinical monitoring. The findings align with the World Health Organization’s programmatic push for decentralized HCV RNA testing shorten time-to-diagnosis and improve linkage to care, particularly in settings where repeated venipuncutre and centralized laboratory access are barriers.NAT testing can pose logistical challenges — the fingerstick workflow proved particularly well-suited for dialysis unit practice allowing bedsite sampling and same-session decision-making. Notably, a substransial propotion of participants were either undergoing direct-acting antiviral (DAA) therapy or had completed treatment, with several achieving sustained virological response (SVR). Among the seven participants with detectable HCV RNA, four had completed DAA therapy and achieved SVR, while the remainder were still on treatment at the time of sampling. This distribution likely explain the predomiance of HCV-RNA-negative results, reflecting susccessful treatment outcome and ongoing virological suppression. Both GeneXpert assays demonstrated strong correlation with the Cobas Taqman 96 (R^2^ values >0.95 for quantifiable results), and high concordance across clinincally relevant timepoints, including pre-treatment, end-of-treatment (EOT) and SVR. Agreement was perfect (Cohen’s κ = 1.000; p < 0.001), with sensitivity, specificity, PPV, and NPV each 100% in this cohort. Comparable results have been reported in different settings. A study conducted in Tanzania, Africa demonstrated excellent correlation concordance between fingerstick and plasma samples, underscoring the clinical utility of this approach in resource-limited environments. Given that most individuals with HCV infection remain undiagnosed, expanding access to this testing modality could be instrumental in expanding access to confirmatory HCV testing and reducing the proportion of undiagnosed cases across Africa [8,16]. Similarly, Bregenzer et al. highlighted the advantages of capillary testing using GeneXpert fingerstick in patient with poor venous access, with diagnostic performance maintained except at very low RNA levels near the limit of quantification (LOQ). In a larger cohort of 111 patients, both GeneXpert HCV VL and Fingerstick assays showed strong correlation with the Cobas Taqman reference (R² = 0.9165 and R² = 0.9899, respectively) and high overall concordance across multiple clinical contexts, including pre-treatment, EOT and SVR. Sensitivity rates of 97.0% for plasma and 100% for fingerstick, with discordant results occurring almost exclusively near the (LOQ), a well-recognixze phenomenon during DAA therapy [17].Grebely et al. further confirmed the robustness of the GeneXpert assays using both venous blood and fingerstick samples. The fingerstick approach maintained excellent diagnostic accuracy, with sensivity of 97.7% and specitivity 99.1% for HCV RNA quantification, reinforcing its feasibility for use resource-limited setting [18]. Wlassow et al. extended validation across all major HCV genotypes (1–6), demonstrating close agreement with Abbott RealTime and Cobas TaqMan assays, with a minimal mean bias of ~0.2 log IU/mL. Importantly, their evaluation of dried blood spots (DBS) achieved 100% sensitivity and >90% specificity, though viral loads were slightly underestimated compared to plasma. These findings emphasize the versatility of the GeneXpert platform across diverse populations and sample types, aligning with WHO recommendations for simplified, decentralized diagnostic strategies. For hemodialysis patients—who often face challenges with venous access and where on-site decision-making is crucial—such approaches may offer practical solutions to strengthen linkage-to-care and accelerate progress toward HCV elimination targets [19]. Building on this evidence, Calvaruso (Italy, 2019) specifically assessed theGeneXpert HCV VL Fingerstick assay for on treatment monitoring during DAA therapy. At Week 4, viral suppression was detected 39 of 56 patients (69.6%) by fingerstick testing and 42 of 56 (75%) by laboratory RT‐PCR. Discordances were confined to very low RNA levels near the LOQ: six patients were undetectable by RT‐PCR yet had < 10 IU/mL on by fingerstick, while five were undetectable by fingerstic but showed <15IU/mL by RT-PCR. By the end of treatment, all patients (100%) were undetectable on both assays, and at SVR 12, both methods identified a single relapse with complete agreement. These results demondemonstrated that testing can reliably monitoring viral kinetics during therapy, with performance comparable to the standard laboratory-based RT-PCR, while also offering the operational advantages of PoC implementation [20]. Overall, our study demonstrated perfect qualitative agreement with the laboratory reference (Cohen’s κ = 1.000; sensitivity, specificity, PPV, and NPV each 100%) and strong quantitative concordance (R^2^ > 0.95). These results support the use of the GeneXpert HCV Fingerstick assay as a practical alternative to plasma-based NAT for most clinical applications, with the caveat of reduced reliability at very low-level RNA levels near the LOQ. Despite the modest sample size, a post-hoc power analysis confirmed that the study was sufficiently powered (observed r = 0.96; 1–β = 1.000), strengthening the validity of the concordance findings in a dialysis-specific population. Nevertheless, this two-center study has inherent limitations. The relatively small cohort and limited geographic scope restrict the precision of diagnostic estimates and generalizability beyond the hemodialysis setting. Dialysis-specific factors-such as variable peripheral perfusion, microvolume sampling, and the intradialytic heparinization to maintain circuit patency- may introduce pre-analytical variability, particularly at very low viral loads, although they do not affect assay’s intrinsic LOQ. Moreover heparin dosing and timing relative to sampling were not systematically documented. Importanly, testing was conducted with analysts blinding to the RT-PCR reference result, which reduce the risk of observer bias. Future multicenter studies with larger cohorts and more diverse populations are warranted to confirm these findsings and assess the long-term utility of fingerstick PoC HCV RNA testing in routine clinical practice.

## Conclusion

The GeneXpert HCV VL Fingerstick assay achieved perfect qualitative concordancewith RT-PCR (K = 1.000) and near-identical quantitative correlation (R^2^ > 0.95) in this dialysis-specific cohort. Its practical advantages-capillary sampling minimal training, and one-hour turnaround—make it a credible substitute for plasma-based NAT in most clinical contexts, though caution is warranted near the the LOQ. Broader access to equall accurate PoCT platforms for other viral hepatitis markers, such as HBV or HAV, would further strengthen viral hepatitis control. More broadly, fingerstick-based HCV RNA testing offers rapid, accessible and less-invasive diagnostic pathway and representing a meaningful advance for underserved populations. Wider implementation, combined with prespecified algorithms for managing low-level-viremia and multicenter validation, would be a positive step toward achieving the World Health Organization’s 2030 HCV elimination targets.

## Supporting information

S1 File STARD 2015 checklist for reporting diagnostic accuracy studies. (DOCX)
